# Isoflavone-Rich Fraction of Traditional Thai Fermented Soybean (Thua Nao) Protects Dermal Fibroblasts from Photoaging by Modulating MAPK and Akt Signaling Pathways

**DOI:** 10.3390/ijms27167303

**Published:** 2026-08-16

**Authors:** Natsinee U-on, Thitikan Jaiwong, Aitsaraphorn Prongjit, Tistaya Semangoen, Jittasak Khowsathit, Pornngarm Dejkriengkraikul, Supachai Yodkeeree

**Affiliations:** 1 Department of Biochemistry, Faculty of Medicine, Chiang Mai University, Chiang Mai 50200, Thailand; natsinee.u@cmu.ac.th (N.U.-o.); thitikanjaiwong656@gmail.com (T.J.); mayissaraporn411@gmail.com (A.P.); jittasak.khowsathit@cmu.ac.th (J.K.); pornngarm.d@cmu.ac.th (P.D.); 2 Department of Medical Technology, Faculty of Allied Health Sciences, Burapha University, Chonburi 20131, Thailand; tistaya@go.buu.ac.th; 3 Anticarcinogenesis and Apoptosis Research Cluster, Faculty of Medicine, Chiang Mai University, Chiang Mai 50200, Thailand

**Keywords:** fermented soybean, human dermal fibroblasts, isoflavones, oxidative stress, photoaging, Thua Nao, ultraviolet B

## Abstract

Ultraviolet B (UVB) irradiation is a major environmental factor contributing to skin photoaging by inducing oxidative stress, apoptosis, inflammation, and extracellular matrix degradation in dermal fibroblasts. This study investigated the photoprotective effects of Thua Nao, a Thai fermented soybean, against UVB-induced human dermal fibroblast damage, and explored its underlying mechanisms. The dichloromethane fraction of Thua Nao (TN-DC) most effectively mitigated UVB-induced cell death. HPLC analysis identified daidzein and glycitein as the major constituent isoflavones in TN-DC that protect fibroblasts against UVB-induced cellular damage. Mechanistically, they reduced apoptosis by suppressing caspase-9 and poly (ADP-ribose) polymerase activation and preserving mitochondrial membrane potential. Additionally, they suppressed inflammatory mediators including interleukin-6, interleukin-8, inducible nitric oxide synthase, and cyclooxygenase-2 and prevented collagen loss. These protective outcomes correlated with decreased intracellular reactive oxygen species and upregulated endogenous antioxidant enzymes including superoxide dismutase 1 and heme oxygenase. Signaling pathway analysis revealed that TN-DC activated the pro-survival extracellular-signal-regulated kinase and Akt pathways in UVB-exposed cells. Conversely, daidzein and glycitein selectively attenuated c-Jun N-terminal kinase activation, downregulating downstream pro-inflammatory cytokines and mediators. Collectively, these findings demonstrate that TN-DC protects human dermal fibroblasts against UVB-induced photoaging primarily by enhancing endogenous antioxidant defense, thereby preserving cellular homeostasis through coordinated regulation of oxidative stress-responsive signaling pathways.

## 1. Introduction

Photoaging is characterized by the progressive deterioration of skin structure and function, resulting in wrinkle formation, loss of elasticity, impaired tissue repair, and visible signs of premature aging. These alterations are closely associated with disruption of extracellular matrix (ECM) homeostasis and progressive degradation of dermal architecture. Dermal fibroblasts are the principal cell type responsible for maintaining ECM integrity through the synthesis and remodeling of structural proteins, particularly collagen. Consequently, fibroblast dysfunction contributes directly to collagen loss, impaired tissue regeneration, and the development of photoaged skin phenotypes [1].

Ultraviolet B (UVB) and ultraviolet A (UVA) radiation are external factors that drive premature skin aging (photoaging), with different mechanisms. UVA is particularly relevant to dermal fibroblasts because it penetrates more deeply and can stimulate oxidative stress and matrix metalloproteinases, which degrade collagen and other extracellular-matrix components. This contributes to wrinkles, dermal thinning, reduced elasticity, and solar elastosis.

Solar ultraviolet B (UVB; 280–320 nm) radiation is a key environmental driver of photoaging. Although the majority of solar UV radiation is absorbed by the epidermis, a biologically relevant fraction of UVB directly penetrates into the upper dermal compartment, specifically targeting papillary dermal fibroblasts located immediately beneath the dermo-epidermal junction [2,3] UVB induces cellular injury through direct DNA damage, forming cyclobutane pyrimidine dimers and 6–4 photoproducts, and an indirect oxidative burst of reactive oxygen species (ROS) [4,5]. This ROS generation disrupts redox homeostasis, leading to lipid peroxidation, apoptotic signaling, and severe mitochondrial dysfunction [6]. Consequently, these damaged fibroblasts trigger the release of pro-inflammatory cytokines and the upregulation of collagen-degrading matrix metalloproteinases (MMPs) while blocking new collagen synthesis. This combined cascade of genetic lesions, mitochondrial failure, and chronic inflammation disrupts the extracellular matrix, serving as the central mechanism behind skin damage and accelerated aging [7,8].

ROS function as signaling molecules that induce the production of pro-inflammatory cytokines, including interleukin-6 (IL-6) and interleukin-8 (IL-8), as well as inflammatory enzymes such as inducible nitric oxide synthase (iNOS) and cyclooxygenase-2 (COX-2), thereby amplifying UVB-induced cellular injury [9]. Concurrently, oxidative stress drives mitochondrial dysfunction, characterized by compromised membrane integrity, altered membrane potential, and the activation of intrinsic apoptotic pathways, leading to progressive fibroblast deterioration [9,10]. These diverse cellular responses are governed by distinct stress-responsive signaling cascades. Specifically, mitogen-activated protein kinases (MAPKs), including extracellular signal-regulated kinase (ERK), c-Jun N-terminal kinase (JNK), and p38, serve as key mediators linking oxidative stress to inflammatory responses, apoptosis, and cellular adaptation. In parallel, the phosphatidylinositol 3-kinase/protein kinase B (PI3K/Akt) pathway plays an essential role in regulating cell survival, redox homeostasis, and stress resistance. Dysregulation of MAPKs and Akt signaling has been implicated in the pathogenesis of UVB-induced skin damage and photoaging, making these pathways attractive molecular targets for the development of photoprotective interventions [11].

Thua Nao, a traditional fermented soybean product widely consumed in Northern Thailand and neighboring regions, represents a rich dietary source of bioactive isoflavones. Our previous study demonstrated that the dichloromethane fraction of Thua Nao (TN-DC) and its primary isoflavones, daidzein and glycitein, significantly protect keratinocytes from UVB-induced oxidative damage and inflammation by modulating MAPKs and Akt signaling [12]. However, the biological activities of Thua Nao-derived fractions against UVB-induced fibroblast damage remain poorly understood. Therefore, the present study aimed to investigate the photoprotective effects of TN-DC and its major isoflavone constituents against UVB-induced damage in human dermal fibroblasts. We specifically characterize the underlying molecular mechanisms, focusing on how TN-DC regulates oxidative stress, inflammation, apoptosis, antioxidant defenses, and collagen synthesis through the MAPK and PI3K/Akt signaling cascades to mitigate photoaging.

## 2. Results

### 2.1. Thua Nao Extract Fractions and Its Major Isoflavones Attenuate UVB-Induced Cytotoxicity in Human Dermal Fibroblasts

**Figure 1:**
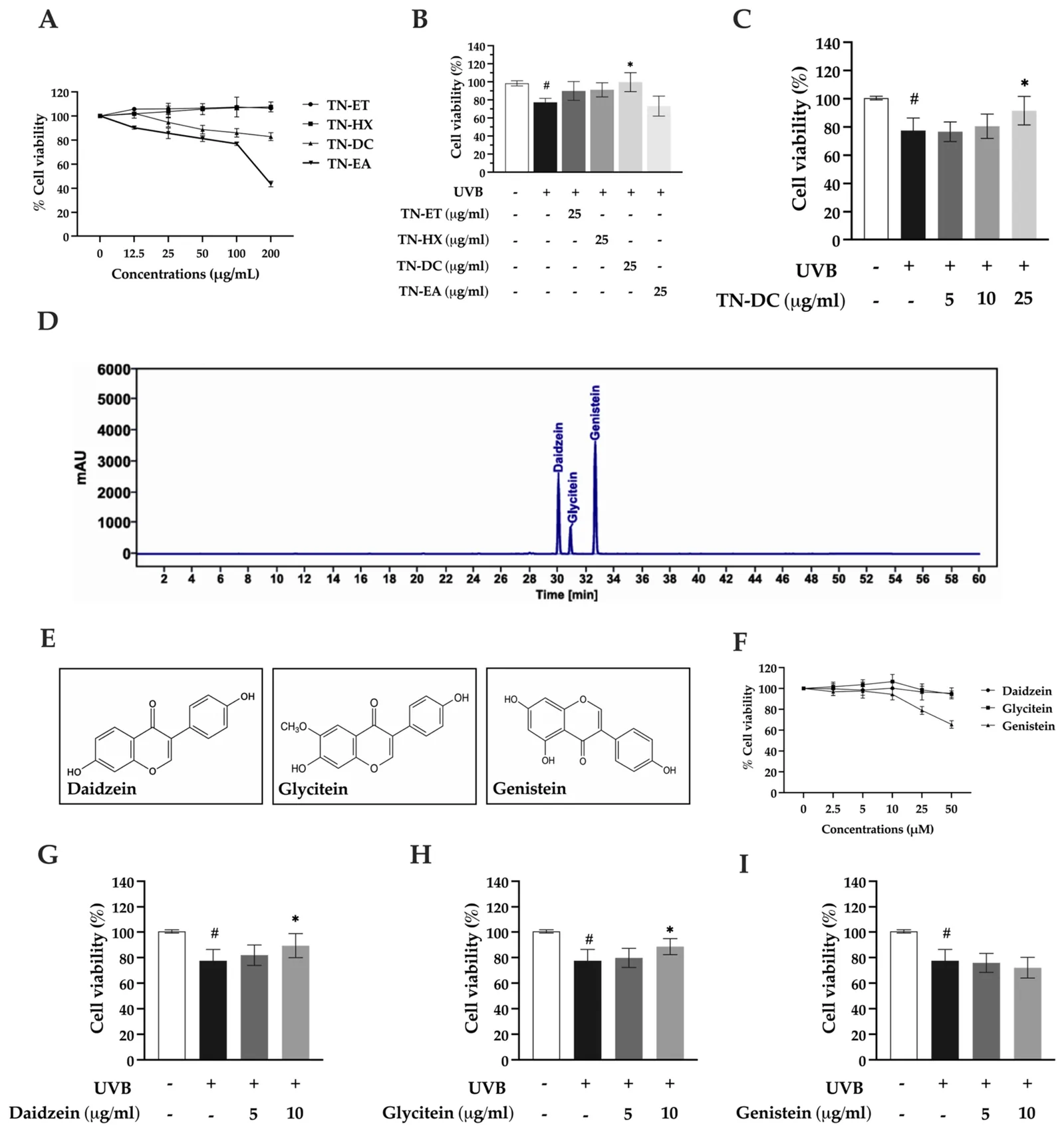
Cytotoxicity of Thua Nao extract fractions, HPLC characterization of the TN-DC fraction, and protective effects of TN-DC and its major isoflavones against UVB-induced cytotoxicity in human dermal fibroblasts. Cytotoxicity of Thua Nao extract fractions, including TN-ET, TN-HX, TN-DC, and TN-EA, determined by the SRB assay after 48 h of treatment (**A**). Protective effect of TN-DC against UVB-induced cytotoxicity in human dermal fibroblasts. The cells were pretreated with the fractions for 6 h, then irradiated with UVB at 30 mJ/cm^2^. After incubation for 48 h, cell viability was measured using the SRB assay (**B**,**C**). Representative HPLC chromatogram of the TN-DC fraction showing the major isoflavones, daidzein, glycitein, and genistein (**D**). Chemical structures of the major isoflavones identified in the TN-DC fraction (**E**). Cytotoxicity of daidzein, glycitein, and genistein determined by the SRB assay after 48 h of treatment (**F**). Protective effects of daidzein (**G**), glycitein (**H**), and genistein (**I**) against UVB-induced cytotoxicity in human dermal fibroblasts. Cell viability was determined using the SRB assay and expressed relative to the untreated control or the UVB-irradiated control group. Data are presented as the mean ± SD from three independent experiments (*n* = 3). # *p* < 0.05 versus the untreated control group; * *p* < 0.05 versus the UVB-irradiated control group.

Before evaluating the protective effects of Thua Nao extract fractions against UVB-induced damage, the cytotoxicity of different Thua Nao extract fractions was first evaluated in human dermal fibroblasts using the SRB assay. As shown in Figure 1A, the ethanolic (TN-ET) and hexane (TN-HX) fractions exhibited minimal cytotoxicity throughout the tested concentration range. In contrast, the ethyl acetate fraction (TN-EA) significantly decreased cell viability in a concentration-dependent manner, reducing viability to below 50% at 200 μg/mL, whereas TN-DC maintained over 80% cell viability at the highest concentration tested.

The protective effect of the Thua Nao extract fractions against UVB-induced cytotoxicity was subsequently evaluated using the SRB assay. As shown in Figure 1B, UVB irradiation at 30 mJ/cm^2^ significantly decreased fibroblast viability to 77.3% compared with the non-irradiated control group. Among the four extract fractions tested at 25 μg/mL, pretreatment with the TN-DC produced the greatest protective effect by significantly restoring cell viability to 99.6% compared to non-UV group. Pretreatment with the TN-HX and TN-ET fractions protected cells from UVB-induced damage, increasing survival rates to 91.1% and 89.9%, respectively, although these increases were not statistically significant. On the other hand, the TN-EA showed no protective effect. Therefore, TN-DC was selected for subsequent experiments. Dose-response analysis demonstrated that TN-DC significantly improved cell viability at 25 μg/mL, whereas lower concentrations (5 and 10 μg/mL) did not produce significant protective effects (Figure 1C). To characterize the bioactive flavonoid constituents within the selected fraction, the HPLC peak profiles of TN-DC were evaluated alongside isoflavone standards (Figure S1). As shown in Figure 1D, three major peaks corresponding to daidzein, glycitein, and genistein were identified, and their chemical structures are presented in Figure 1E. The concentrations of daidzein, glycitein, and genistein in TN-DC were 41.28, 21.25, and 72.57 µg/mg of extract, respectively (Table 1). The cytotoxicity and protective effects of the major isoflavones identified in TN-DC were subsequently evaluated. As shown in Figure 1F, daidzein and glycitein exhibited minimal cytotoxicity over the tested concentration range. In contrast, genistein reduced fibroblast viability at higher concentrations, decreasing cell viability to nearly 60% at 50 μg/mL. Under UVB-irradiated conditions, pretreatment with daidzein and glycitein significantly restored cell viability at 10 μg/mL (Figure 1G,H), whereas genistein did not significantly protect fibroblasts against UVB-induced cytotoxicity (Figure 1I). These findings suggest that daidzein and glycitein are the bioactive isoflavones contributing to the cytoprotective activity of the TN-DC fraction.

### 2.2. TN-DC and Its Active Compounds Preserve Mitochondrial Integrity and Attenuate UVB-Induced Apoptosis in Human Dermal Fibroblasts

**Figure 2:**
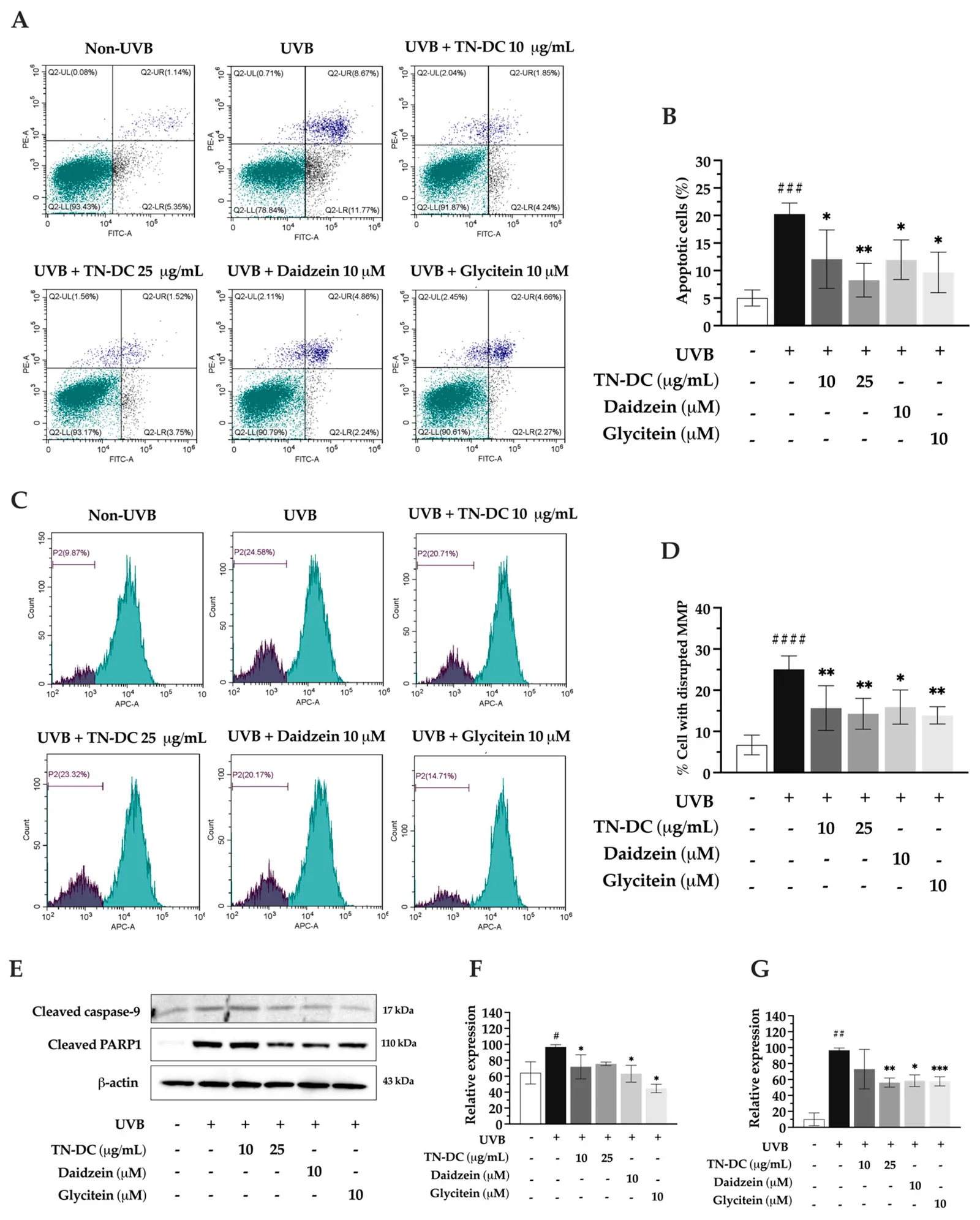
TN-DC and its major isoflavones suppress UVB-induced mitochondrial dysfunction and intrinsic apoptotic signaling in human dermal fibroblasts. The cells were pretreated with the TN-DC or active isoflavones for 6 h, then irradiated with UVB at 30 mJ/cm^2^ after incubation for 24 h. Representative flow cytometric dot plots of Annexin V-FITC/PI staining showing apoptotic cell populations in human dermal fibroblasts following UVB irradiation and pretreatment with TN-DC, daidzein, or glycitein (green LL means survival cells, gray LR means early apoptotic cells, dark purple UR means late apoptotic cells, and dark purple UL means necrosis cells) (**A**). Quantitative analysis of apoptotic cells determined by flow cytometry (**B**). Representative flow cytometric histograms showing MMP determined using the MitoView™ 633 (dark purple means disrupted MMP population, and green means normal MMP population) (**C**). Quantitative analysis of cells with disrupted MMP (**D**). Representative Western blot images of cleaved caspase-9 and cleaved PARP1 protein expression. β-actin was used as the loading control (**E**). Densitometric analysis of cleaved caspase-9 (**F**) and cleaved PARP1 (**G**) protein expression normalized to β-actin. Data are presented as the mean ± SD from three independent experiments (*n* = 3). # *p* < 0.05, ## *p* < 0.01, ### *p* < 0.001, and #### *p* < 0.0001 versus the non-UVB control group; * *p* < 0.05, ** *p* < 0.01, and *** *p* < 0.001 versus the UVB-treated group.

To determine whether the cytoprotective effect of TN-DC was associated with the suppression of apoptosis, UVB-induced apoptosis was evaluated by Annexin V-FITC/PI staining and flow cytometry. As shown in Figure 2A,B, UVB irradiation markedly increased the percentage of apoptotic cells to 20.27% compared with the non-irradiated control group. Pretreatment with TN-DC (10 and 25 µg/mL) significantly reduced the percentage of apoptotic cells to 12.08% and 8.27%, respectively. Similarly, pretreatment with daidzein or glycitein (10 µM) significantly decreased the percentage of apoptotic cells to 11.97% and 9.66%, respectively. Because mitochondrial dysfunction is a hallmark of intrinsic apoptosis, mitochondrial membrane potential (MMP) was subsequently evaluated using the MitoView™ 633. Representative flow cytometric histograms are shown in Figure 2C,D. UVB irradiation significantly increased the percentage of cells with disrupted MMP to 25.0% compared with the non-irradiated control group. Pretreatment with TN-DC, daidzein, or glycitein effectively prevented the loss of MMP in UVB-irradiated fibroblasts. Specifically, TN-DC (10 and 25 μg/mL), daidzein (10 μM), and glycitein (10 μM) significantly decreased the percentage of cells with disrupted MMP to 15.6%, 14.2%, 15.9%, and 13.8%, respectively, compared with the UVB-treated group. To determine whether the observed preservation of mitochondrial function was accompanied by modulation of apoptotic signaling, the expression of cleaved caspase-9 and cleaved PARP1 was examined by Western blot analysis. As shown in Figure 2E–G, UVB irradiation significantly increased the protein expression of cleaved caspase-9 and cleaved PARP1 compared with the non-irradiated control. Pretreatment with TN-DC significantly suppressed the UVB-induced cleavage of both caspase-9 (Figure 2F) and PARP1 (Figure 2G). Similarly, daidzein and glycitein significantly reduced the expression of these apoptotic proteins, consistent with the flow cytometric findings. Collectively, these findings indicate that TN-DC, daidzein and glycitein protect human dermal fibroblasts against UVB-induced apoptosis by preserving mitochondrial function and suppressing intrinsic apoptosis-related signaling.

### 2.3. TN-DC Suppresses UVB-Induced Inflammatory Responses and Preserves Collagen Content in Human Dermal Fibroblasts

**Figure 3:**
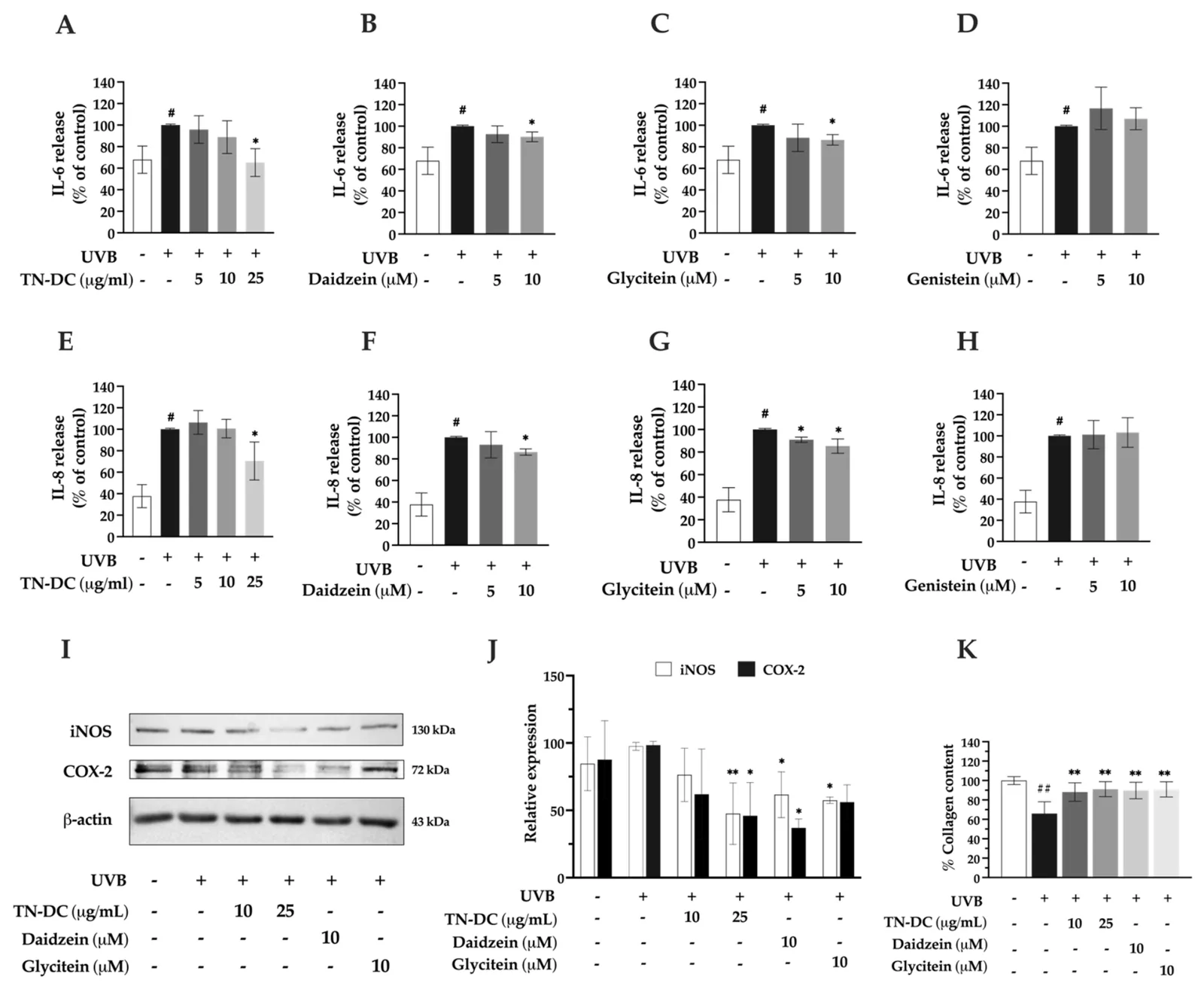
TN-DC and its major isoflavones suppress UVB-induced inflammatory responses and preserve collagen content in dermal fibroblast cells. The cells were pretreated with TN-DC, daidzein, glycitein, or genistein for 6 h prior to UVB irradiation (20 mJ/cm^2^). After 18 h, IL-6 and IL-8 levels in the culture supernatants were quantified by ELISA. Effects of TN-DC, daidzein, glycitein, and genistein on UVB-induced IL-6 release (**A**–**D**). Effects of TN-DC, daidzein, glycitein, and genistein on UVB-induced IL-8 release (**E**–**H**). The cells were pretreated with TN-DC, daidzein, and glycitein for 6 h prior to UVB irradiation (20 mJ/cm^2^). After incubation for 18 h, iNOS and COX-2 protein expressions were determined by Western blot analysis. Representative Western blot images of iNOS and COX-2 protein expression. β-actin was used as the loading control (**I**). Densitometric analysis of iNOS and COX-2 protein expression normalized to β-actin (**J**). (**K**) Collagen content was measured using the Sircol™ soluble collagen assay. Data are presented as the mean ± SD from three independent experiments (*n* = 3). # *p* < 0.05 and ## *p* < 0.01 versus the non-irradiated control group; * *p* < 0.05 and ** *p* < 0.01 versus the UVB-irradiated group.

To determine whether TN-DC suppresses UVB-induced inflammatory responses, the production of the pro-inflammatory cytokines IL-6 and IL-8 was first examined. As shown in Figure 3A–H, UVB irradiation significantly increased the release of both IL-6 and IL-8 compared with the non-irradiated control group. Pretreatment with TN-DC significantly reduced the production of both cytokines at 25 μg/mL, whereas lower concentrations (5 and 10 μg/mL) did not show significant inhibitory effects. Similarly, pretreatment with daidzein (10 μM) or glycitein (10 μM) significantly decreased the release of both IL-6 and IL-8. In contrast, genistein did not significantly affect the production of either cytokine. The effects of TN-DC, daidzein, and glycitein on the downstream inflammatory mediators iNOS and COX-2 were subsequently examined by Western blot analysis. As shown in Figure 3I,J, pretreatment with TN-DC significantly reduced the expression of iNOS and COX-2. Similar inhibitory effects were observed following pretreatment with daidzein, whereas glycitein only downregulated the expression of iNOS. During inflammaging, proinflammatory cytokines impair fibroblast activity by downregulating collagen synthesis and accelerating extracellular matrix degradation. Therefore, we quantified soluble collagen content using the Sircol™ assay to evaluate the protective effects of TN-DC and its active isoflavones. As shown in Figure 3K, UVB irradiation significantly decreased collagen content compared with the non-irradiated control group. Pretreatment with TN-DC, daidzein, and glycitein significantly preserved collagen content following UVB exposure. Additionally, the secretion level of MMP-2 from dermal fibroblast was evaluated by gelatin zymography. As shown in Figure S2, pretreatment with TN-DC or its major isoflavones did not significantly suppress the secretion level of MMP-2. Collectively, these findings demonstrate that TN-DC attenuates UVB-induced inflammatory responses and preserves collagen content in human dermal fibroblasts.

### 2.4. TN-DC and Its Major Isoflavones Attenuate UVB-Induced Intracellular ROS Generation and Enhance Antioxidant Protein Expression in Human Dermal Fibroblasts

**Figure 4:**
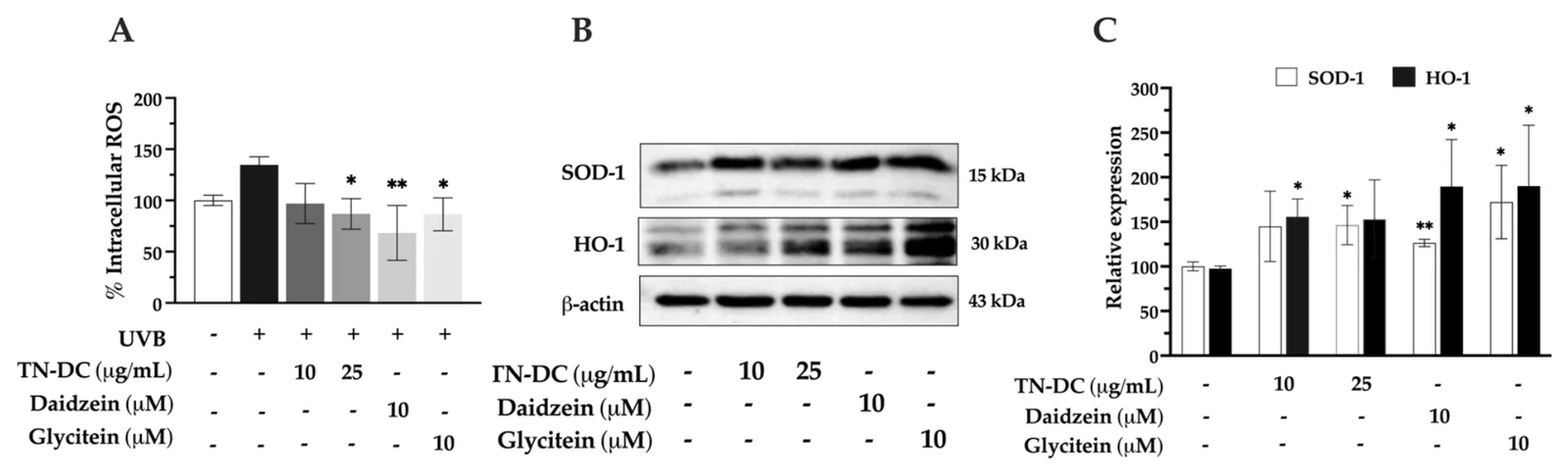
TN-DC and its major isoflavones suppress UVB-induced oxidative stress and enhance antioxidant protein expression in human dermal fibroblasts. Intracellular reactive oxygen species (ROS) levels were measured following UVB irradiation in the presence or absence of TN-DC, daidzein, or glycitein (**A**). The cells were pretreated with TN-DC and active isoflavones for 6 h and incubated with DCF-DA for 30 min before being UVB irradiated (at 20 mJ/cm^2^). After UVB exposure for 1 h, the fluorescence was immediately measured at an excitation/emission of 485/525 nm using a fluorescence microplate reader. ROS levels are expressed as the percentage of the non-irradiated control. The cells were treated with the fractions for 48 h. After treatment, SOD-1 and HO-1 protein expression was determined by Western blot analysis (**B**). Representative Western blot images showing the protein expression of SOD-1 and HO-1, with β-actin used as the loading control (**B**). Densitometric analysis of SOD-1 and HO-1 protein expression normalized to β-actin and expressed relative to the non-irradiated control (**C**). Data are presented as the mean ± SD (*n* = 3 independent experiments). * *p* < 0.05 and ** *p* < 0.01 versus the UVB-treated group.

The free radical scavenging activities of TN-DC and its major isoflavones were first evaluated using the DPPH and ABTS assays. As summarized in Table 2, TN-DC exhibited a DPPH IC_50_ value of 5.42 mg/mL, whereas daidzein and glycitein showed IC_50_ values of 57.01 and 46.24 mM, respectively. Vitamin E, used as the positive control, exhibited a DPPH IC_50_ value of 0.003 mg/mL. Similarly, in the ABTS assay, TN-DC, daidzein, and glycitein exhibited IC_50_ values of 0.97 mg/mL, 0.21 mM, and 0.21 mM, respectively, whereas the positive control, Trolox, exhibited an IC_50_ value of 0.005 mg/mL. To evaluate the effects of TN-DC and its major isoflavones on UVB-induced cellular oxidative stress, intracellular ROS levels were determined in human dermal fibroblasts. As shown in Figure 4A, UVB irradiation significantly increased intracellular ROS production to 134.76% compared with the non-irradiated control group. Pretreatment with TN-DC (25 μg/mL), daidzein (10 μM), or glycitein (10 μM) significantly suppressed UVB-induced ROS accumulation to 86.86%, 68.28%, and 86.49%, respectively. To further explore the antioxidant effects of TN-DC and its major isoflavones, the protein expression of the endogenous antioxidant enzymes SOD-1 and HO-1 was examined by Western blot analysis. Representative immunoblots are shown in Figure 4B, and densitometric analysis is presented in Figure 4C. Pretreatment with TN-DC increased the expression of both SOD-1 and HO-1, although the increase in HO-1 was significant only at 10 μg/mL. Daidzein and glycitein significantly enhanced the expression of both antioxidant proteins.

### 2.5. TN-DC Modulates the Phosphorylation of MAPKs and Akt in UVB-Irradiated Human Dermal Fibroblasts

**Figure 5:**
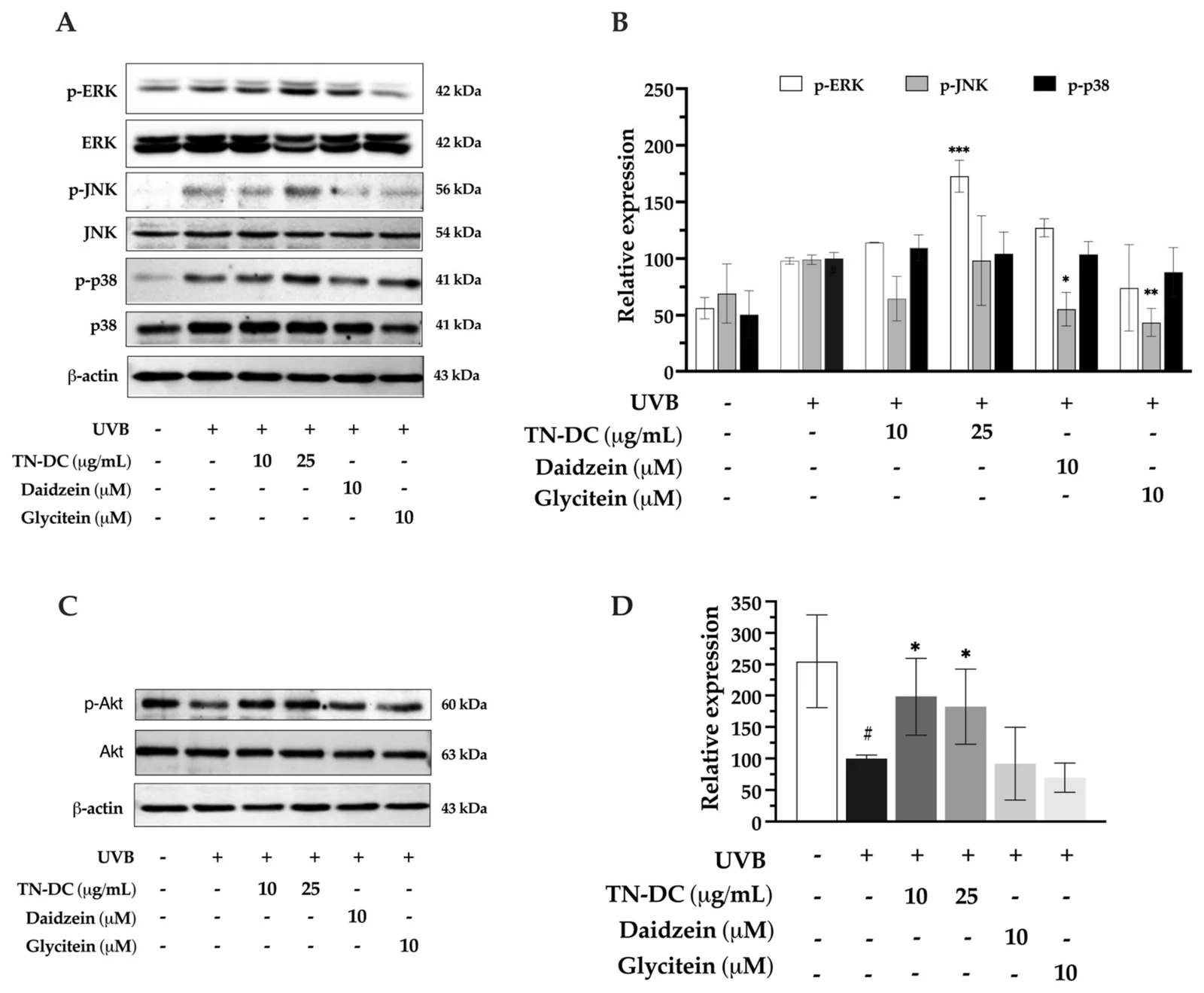
Effects of TN-DC and its major isoflavones on the phosphorylation of MAPKs and Akt in UVB-irradiated human dermal fibroblasts. Representative Western blots showing the phosphorylation of ERK, JNK, and p38, together with their corresponding total protein levels, are presented in (**A**), and the densitometric analysis of phosphorylated MAPKs is shown in (**B**). Representative Western blots showing the phosphorylation of Akt and total Akt expression are presented in (**C**), and the corresponding densitometric analysis is shown in (**D**). The cells were pretreated with TN-DC (10 or 25 μg/mL), daidzein (10 μM), or glycitein (10 μM) for 6 h prior to UVB irradiation (30 mJ/cm^2^), followed by incubation for an additional 1 h. Phosphorylated protein levels were quantified by densitometry, normalized to β-actin, and expressed relative to the UVB-treated control group. Data are presented as mean ± SD from three independent experiments (*n* = 3). # *p* < 0.05 versus the non-irradiated control group; * *p* < 0.05, ** *p* < 0.01, and *** *p* < 0.001 versus the UVB-treated group.

To investigate whether the photoprotective and anti-inflammatory properties of TN-DC and its active isoflavones involve stress-responsive signaling, the phosphorylation of MAPKs (ERK, JNK, and p38) and Akt was evaluated via Western blot analysis. Representative immunoblots and the corresponding densitometric analysis of MAPKs are shown in Figure 5A,B, respectively. UVB irradiation increased the phosphorylation of ERK, JNK, and p38 compared with the non-irradiated control group. Pretreatment with TN-DC (10 μg/mL) attenuated JNK phosphorylation but was not significant, while a higher dose (25 μg/mL) significantly upregulated ERK phosphorylation. In contrast, daidzein and glycitein treatment (10 μM) reduced JNK activation. Conversely, none of the treatments induced significant changes in p38 phosphorylation. The modulation effects of TN-DC and its major isoflavones on Akt phosphorylation were subsequently examined. As shown in Figure 5C,D, UVB irradiation markedly reduced Akt phosphorylation compared with the non-irradiated control group. Pretreatment with TN-DC at both 10 and 25 μg/mL significantly restored Akt phosphorylation, whereas daidzein and glycitein did not significantly alter Akt phosphorylation. Collectively, these findings demonstrate that TN-DC modulates the phosphorylation of MAPKs and Akt in UVB-irradiated human dermal fibroblasts.

## 3. Discussion

Both UVA and UVB radiation are primary environmental drivers of premature skin aging (photoaging), though they act via distinct mechanisms and penetrate to different dermal depths [3]. While UVA constitutes approximately 95% of terrestrial UV radiation and reaches the deeper reticular dermis, solar UVB (280–320 nm) carries significantly higher photon energy per unit area. Although the majority of UVB is absorbed by the epidermis, a biologically relevant fraction (~5–10%) penetrates beyond the dermo-epidermal junction to directly target resident fibroblasts within the upper papillary dermis [13]. This exposure initiates direct photochemical DNA damage (such as cyclobutane pyrimidine dimer formation) and triggers matrix metalloproteinase-driven degradation cascades. Therefore, investigating low-dose UVB in papillary fibroblasts provides critical mechanistic insights into direct, high-energy photochemical stress within the superficial dermis, complementing the predominantly oxidative pathways driven by UVA. Because dermal fibroblasts are the principal cells responsible for extracellular matrix maintenance, collagen synthesis, and tissue repair, their dysfunction represents a critical event in the initiation and progression of photoaging. Consequently, preserving fibroblast homeostasis has emerged as an important strategy for preventing UVB-induced skin damage [14]. Although numerous natural products have been reported to exert antioxidant or anti-inflammatory activities against UV-induced skin damage, relatively few studies have comprehensively investigated whether fermented food-derived phytochemicals can coordinately protect multiple biological processes involved in fibroblast dysfunction during photoaging [9]. In the present study, we demonstrate that the TN-DC exerts photoprotective effects through the coordinated regulation of several interconnected cellular responses associated with UVB-induced injury. Rather than acting on a single pathological event, TN-DC preserved fibroblast viability while simultaneously attenuating oxidative stress, mitochondrial dysfunction, inflammatory responses, and extracellular matrix deterioration, suggesting a broader role in maintaining fibroblast homeostasis under UVB-induced stress.

The photoprotective effects of TN-DC are likely attributable, at least in part, to the unique phytochemical composition generated during soybean fermentation. Fermentation has been widely reported to promote the hydrolysis of isoflavone glycosides into their corresponding aglycone forms through the action of microbial β-glucosidases, thereby enhancing their bioavailability and biological activities. Because of their lower molecular weight and higher lipophilicity, aglycone isoflavones cross cell membranes and penetrate the stratum corneum significantly faster and more efficiently than their glycoside counterparts. This structural advantage directly correlates with improved topical absorption and enhanced cellular uptake in dermal layers. In our previous study, TN-DC exhibited the greatest photoprotective activity among all Thua Nao fractions in UVB-irradiated HaCaT keratinocytes, suggesting that this fraction contains bioactive constituents responsible for protection against UVB-induced cellular damage [12]. Consistent with these observations, HPLC analysis in the present study identified daidzein, glycitein, and genistein as the predominant aglycone isoflavones in the TN-DC fraction, indicating that microbial fermentation substantially reshaped the phytochemical profile of Thua Nao.

The initial step of photoaging involves the loss of fibroblast viability following UVB exposure. Consistent with this mechanism, UVB irradiation significantly reduced fibroblast survival, whereas TN-DC pretreatment markedly restored cell viability. Interestingly, despite sharing a similar aglycone backbone, only daidzein and glycitein significantly protected the cells against UVB-induced cytotoxicity, whereas genistein failed to exhibit comparable cytoprotective activity. This variation aligns with prior studies reporting that daidzein and glycitein effectively mitigate UVB-induced skin damage, whereas low concentrations of genistein exert no inhibitory effect on dermal fibroblast death. This observation suggests that relatively small structural differences, particularly the number and position of hydroxyl and methoxy substituents, can markedly influence the physicochemical properties, antioxidant capacity, membrane permeability, and biological activities of flavonoids and isoflavones [15,16]. Structurally, polyhydroxy flavones follow a specific deprotonation energy sequence: 7-OH > 4′-OH > 5-OH [17]. Consequently, the 5-OH group present in genistein is highly resistant to deprotonation, which diminishes its radical-scavenging potential. This thermodynamic constraint explains why daidzein effectively prevents UVB-induced DNA damage while genistein at the same concentration fails to confer comparable protection [18]. Taken together, these findings imply that the 5-OH group in genistein hinders its photoprotective efficacy, whereas the structural configurations of daidzein and glycitein favor cellular preservation. Interestingly, although daidzein and glycitein reproduced many of the protective effects of TN-DC, the whole TN-DC fraction generally exhibited comparable or greater efficacy across multiple experimental endpoints. This observation suggests that the biological activity of TN-DC cannot be fully attributed to individual isoflavones alone. Instead, additional phytochemicals present in the dichloromethane fraction may interact synergistically with the major aglycone isoflavones to enhance the overall photoprotective activity. Such synergistic interactions among multiple phytochemicals have frequently been reported for botanical extracts and fermented foods, in which minor constituents collectively contribute to greater biological efficacy than isolated compounds [19,20].

UVB radiation initiates skin photodamage by targeting epidermal keratinocytes and dermal fibroblasts to trigger apoptosis. This programmed cell death is driven by direct DNA mutations, mitochondrial membrane disruption, and oxidative stress. Consequently, agents that suppress UVB-induced apoptosis directly enhance photoprotection. Here, we demonstrate that TN-DC, daidzein, and glycitein effectively rescue dermal fibroblasts from this apoptotic cell death. UVB-induced oxidative stress fundamentally disrupts mitochondrial outer membrane integrity, leading to the loss of MMP. This permeabilization prompts the cytosolic release of cytochrome c, which binds to apoptotic protease activating factor-1 to form the apoptosome complex, subsequently recruiting and activating the initiator procaspase-9. Once active, caspase-9 cleaves and activates the downstream executioner caspase-3, which propagates the apoptotic cascade by cleaving essential structural and regulatory proteins, including PARP1. Consequently, PARP1 cleavage facilitates cellular disassembly, serving as a definitive biochemical hallmark of UV-induced apoptosis. Consistent with previous reports, our study demonstrates that UVB irradiation induced MMP disruption in fibroblast cells. Crucially, treatment with TN-DC, daidzein, or glycitein significantly mitigated this mitochondrial collapse while simultaneously downregulating the expression of cleaved caspase-9 and PARP1. These findings indicate that TN-DC, daidzein, and glycitein protect dermal fibroblasts from UVB-induced apoptosis by suppressing the mitochondrial apoptotic pathway.

Excessive UVB exposure triggers oxidative stress in the skin, causing dermal fibroblasts to release pro-inflammatory cytokines and mediators. This cascade establishes a destructive feedback loop that accelerates cellular senescence, increases melanin production, and breaks down the extracellular matrix to cause premature skin aging. Our experimental data confirm that UVB irradiation markedly upregulates the expression of IL-6 and IL-8 in dermal fibroblasts. Importantly, treatment with TN-DC, daidzein, or glycitein significantly downregulated IL-6, IL-8, Cox-2 and iNOS expression in these UVB-irradiated fibroblasts. Moreover, elevated pro-inflammatory cytokines and mediators stimulate extracellular matrix (ECM) degradation by upregulating matrix metalloproteinases (MMPs) while simultaneously suppressing procollagen synthesis in fibroblasts, ultimately depleting collagen content and driving skin photoaging [21,22]. Our findings demonstrate that TN-DC, daidzein, and glycitein effectively restore collagen content in UVB-irradiated dermal fibroblasts. Type I collagen forms the core structural framework of the dermal extracellular matrix, imparting tensile strength and firmness to the skin. Its degradation occurs sequentially, wherein intact triple-helical collagen is first cleaved by MMP-1 into fragments that are subsequently degraded by gelatinases like MMP-2 [23]. Although MMP-1 was not directly measured in this study, treatment with TN-DC or its major isoflavones did not attenuate UVB-induced MMP-2 levels. This demonstrates that collagen preservation occurred independently of MMP-2 inhibition. Taken together, this suggests that TN-DC maintains ECM structural integrity primarily by protecting fibroblast homeostasis and promoting anabolic collagen synthesis rather than suppressing gelatinase-mediated degradation.

Oxidative stress is widely recognized as one of the earliest events in UVB-induced photoaging, initiating a cascade of pathological responses that include mitochondrial dysfunction, inflammatory signaling, extracellular matrix degradation, and ultimately fibroblast dysfunction [24]. Therefore, limiting excessive ROS accumulation represents a fundamental strategy for preserving fibroblast function during UVB exposure. In the present study, TN-DC markedly suppressed intracellular ROS generation despite exhibiting modest free radical-scavenging activity in the cell-free DPPH and ABTS assays. This apparent discrepancy suggests that the antioxidant effects of TN-DC cannot be fully explained by direct radical scavenging alone. Instead, the increased expression of SOD-1 and HO-1 observed following TN-DC treatment is consistent with the possibility that TN-DC modulates endogenous antioxidant responses, thereby contributing to the attenuation of intracellular oxidative stress. Similar observations have been reported for several plant-derived phytochemicals, in which protection against UVB-induced oxidative injury was more closely associated with the activation of cellular antioxidant defense systems than with direct chemical antioxidant capacity [25]. Collectively, these findings suggest that the photoprotective activity of TN-DC is primarily associated with its ability to improve cellular redox balance rather than functioning solely as a direct free radical scavenger. This distinction between cell-free antioxidant capacity and intracellular antioxidant activity is increasingly recognized in phytochemical research. Chemical assays such as DPPH and ABTS primarily evaluate the direct electron- or hydrogen-donating ability of compounds under simplified experimental conditions, whereas intracellular ROS levels are determined by a complex balance between ROS generation, mitochondrial function, antioxidant enzyme activity, and cellular redox signaling. Consequently, phytochemicals with relatively weak radical-scavenging activity may nevertheless exert potent antioxidant effects in living cells by suppressing ROS production or enhancing endogenous antioxidant defense systems rather than directly neutralizing free radicals [26,27].

The exposure to UVB radiation generates ROS that activate multiple intracellular cascades, particularly the MAPK and PI3K/Akt signaling pathways. This signaling axis regulates downstream cellular survival, mitochondrial function, and inflammatory cascades, directly influencing collagen synthesis and extracellular matrix turnover to drive dermal photoaging [10]. Within these cascades, sustained JNK and p38 activation drives oxidative stress-mediated apoptosis and inflammatory signaling in UV-exposed cells [28,29]. Upon UVB irradiation, phosphorylated JNK and p38 initiate the intrinsic mitochondrial apoptotic pathway by modulating Bcl-2 family proteins, specifically phosphorylating pro-apoptotic Bax and suppressing anti-apoptotic Bcl-2. This molecular shift prompts the downstream release of cytochrome c, which sequentially activates caspases-9 and -3 to execute apoptosis [29,30]. Moreover, UVB irradiation generates ROS that trigger JNK and p38 MAPK pathway activation in dermal fibroblasts, subsequently driving the inflammatory response. Our study indicates that TN-DC, daidzein and glycitein attenuate UVB-induced JNK phosphorylation without altering p38 activation [31]. This modulation highlights the potential of daidzein, glycitein, and TN-DC to mitigate cellular damage and inflammation primarily via the JNK signaling cascade.

In contrast, activation of ERK and PI3K/Akt pathway promotes cell survival and enhances cellular adaptation to oxidative stress [29,32]. These signaling pathways therefore serve as important molecular regulators linking oxidative stress to downstream biological responses [33,34]. Consistent with this, our findings demonstrate that TN-DC significantly increases phosphorylated ERK and Akt, whereas daidzein and glycitein did not. Furthermore, activating the ERK and PI3K/Akt signaling pathways upregulates key antioxidant enzymes (SOD, HO-1, and GPx4) and drives the synthesis of extracellular matrix proteins, including collagen types I and III, in dermal fibroblasts [24,29,32,33]. Although pathway-specific inhibitors were not utilized in the present fibroblast model, the protective involvement of these survival cascades is well supported by the literature. Previous studies in UVB-irradiated dermal fibroblasts have demonstrated that pharmacological inhibition of Akt (via LY294002) or ERK (via U0126) significantly reduces cell viability, attenuates Nrf2-mediated glutathione synthesis, and suppresses fibroblast proliferation [29,35]. Moreover, our prior work confirmed that specific inhibitors of Akt and ERK completely reversed the cytoprotective effects of TN-DC against UVB-induced HaCaT keratinocyte death [12]. These findings suggest that the activation of ERK and PI3K/Akt signaling by TN-DC may enhancing antioxidant defenses and collagen synthesis, thereby restoring cellular homeostasis and mitigating dermal photoaging.

## 4. Materials and Methods

### 4.1. Chemicals and Reagents

Dulbecco’s modified Eagle medium (DMEM), penicillin/streptomycin, and trypsin EDTA were purchased from Gibco (Grand Island, NY, USA). Fetal bovine serum (FBS) was supplied by Hyclone (Logan, UT, USA). MitoView^TM^ 633 was purchased from Biotium (Fremont, CA, USA). The IL-6 and IL-8 ELISA kits and FITC Annexin V kit were obtained from BioLegend (San Diego, CA, USA). LY294002, PD98059, antibodies specific to β-actin, COX-2, ERK, p38, p44/42 (ERK), p-p38, and p-p44/42 (ERK) were obtained from Cell Signaling Technology (Danvers, MA, USA). Antibodies specific to active caspase-3, caspase-9, PARP1, p-Akt, Akt, p-JNK, JNK, HO-1, iNOS and SOD1 were purchased from Abclonal (Woburn, MA, USA). The DCF-DA was obtained from Sigma (St. Louis, MO, USA). HPLC standards including genistein, daidzein, glycitein, genistin, daidzin, and glycitin (all with HPLC purity > 98%) were obtained from Biopurify Phytochemicals (Chengdu, China). Nitrocellulose membrane and ECL reagent were supplied by GE Healthcare (Little Chalfont, UK). Sulforhodamine B (SRB) was purchased from Sigma (Shanghai, China).

### 4.2. Plant Materials and Sample Preparation

Thua Nao was prepared according to the traditional fermentation process. Briefly, 1 kg of soybeans was soaked in water for 12 h, followed by boiling for 3 h. After cooling to approximately 40 °C, the cooked soybeans were naturally fermented at 37 °C under 80% relative humidity for 3 days. The fermented soybeans were then dried in a hot-air oven at 50 °C and ground into a fine powder. For extraction, 500 g of the powdered sample was extracted twice with 80% (*v*/*v*) ethanol at room temperature overnight. The combined extracts were filtered, concentrated under reduced pressure using a rotary evaporator, and freeze-dried to obtain the crude ethanolic extract (TN-ET). The TN-ET extract was subsequently subjected to liquid–liquid partitioning with organic solvents of increasing polarity, namely hexane, dichloromethane, and ethyl acetate. Each solvent fraction was concentrated under reduced pressure and dried to yield the hexane fraction (TN-HX), dichloromethane fraction (TN-DC), and ethyl acetate fraction (TN-EA), respectively.

### 4.3. HPLC Analysis of TN-DC

TN-DC was dissolved in ethanol at a concentration of 10 mg/mL and filtered through a 0.45 μm syringe filter prior to analysis. An aliquot (5 μL) of the filtered sample was injected into a Zorbax Eclipse Plus C18 reversed-phase column (4.6 × 250 mm, 5 μm; Agilent Technologies, Santa Clara, CA, USA). Chromatographic separation was performed at a flow rate of 1.0 mL/min using a binary mobile phase consisting of 0.1% (*v*/*v*) trifluoroacetic acid in water (solvent A) and methanol (solvent B). Gradient elution was carried out by decreasing solvent A from 100% to 0% while simultaneously increasing solvent B from 0% to 100% over 50 min, followed by an isocratic elution with 100% methanol until 60 min. Chromatograms were recorded using a UV detector at 254, 260, 280, and 320 nm. The chromatographic fingerprints of TN-DC were compared with authentic reference standards commonly identified in fermented soybean products, including genistein, daidzein, glycitein, genistin, daidzin, and glycitin. Isoflavones were identified based on their retention times and UV spectra relative to the corresponding standards. Quantitative analysis was performed by integrating the chromatographic peak areas and calculating the concentrations from external calibration curves prepared using the respective reference compounds.

### 4.4. ABTS (2,2′-Azino-Bis-(Ethylbenthiazoline-6-Sulfonic Acid) Assay

The ABTS (Sigma-Aldrich, Oakville, ON, Canada) radical scavenging activity of TN-DC was determined using the ABTS assay as previously described with slight modifications [36]. Briefly, the ABTS radical cation (ABTS•^+^) was generated by mixing 7 mM ABTS solution with 2.45 mM potassium persulfate (K_2_S_2_O_8_) at a 1:1 (*v*/*v*) ratio. The reaction mixture was incubated in the dark at room temperature for 12–16 h to allow complete radical formation. Prior to analysis, the ABTS•^+^ solution was diluted to the appropriate working concentration. Subsequently, 10 μL of TN-DC at various concentrations or Trolox (Sigma-Aldrich, Brøndby, Denmark), used as the positive control, was mixed with 990 μL of the diluted ABTS•^+^ solution and incubated in the dark for 6 min at room temperature. The absorbance was then measured at 734 nm using a spectrophotometer. The radical scavenging activity was expressed as the percentage inhibition relative to the control, and the half-maximal inhibitory concentration (IC_50_) was calculated from the concentration–response curve.

### 4.5. DPPH (2,2-Diphenyl-1-picryldrazal) Assay

The free radical scavenging activity of TN-DC was evaluated using the 2,2-diphenyl-1-picrylhydrazyl (DPPH) assay with slight modifications from previously reported methods [37,38]. This assay is based on the reduction in the stable DPPH radical by hydrogen- or electron-donating antioxidants, resulting in a decrease in absorbance. Briefly, the DPPH solution was prepared by dissolving 2,2-diphenyl-1-picrylhydrazyl (DPPH; Sigma-Aldrich, Steinheim, Germany) in methanol and diluted to the appropriate working concentration prior to use. TN-DC at various concentrations or α-tocopherol (Sigma-Aldrich, Steinheim, Germany), used as the positive control, was mixed with 180 μL of the diluted DPPH solution and incubated under light-protected conditions for the appropriate reaction time. The absorbance was then measured at 540 nm using a microplate reader. The radical scavenging activity was expressed as the percentage inhibition relative to the control, and the half-maximal inhibitory concentration (IC_50_) was determined from the concentration–response curve.

### 4.6. Cell Culture

Human dermal fibroblasts were purchased from the American Type Culture Collection (ATCC, Manassas, VA, USA; Catalog No. PCS-201-012) and cultured in Dulbecco’s Modified Eagle Medium (DMEM) supplemented with 10% (*v*/*v*) fetal bovine serum (FBS) and 1% (*v*/*v*) penicillin–streptomycin. Cells were maintained at 37 °C in a humidified atmosphere containing 5% CO_2_. For all experiments, TN-DC and the individual isoflavones were dissolved in dimethyl sulfoxide (DMSO; Fluka, Buchs, Switzerland) to prepare stock solutions and subsequently diluted with complete culture medium to the desired concentrations. The final concentration of DMSO was maintained below 0.5% (*v*/*v*) in all treatment groups. Vehicle control cells received an equivalent concentration of DMSO. Cells were used at passages 4–15 to avoid passage-dependent senescence.

### 4.7. Cell Viability Assay

Cell viability was determined using the sulforhodamine B (SRB) assay. Human dermal fibroblasts were seeded in 96-well plates at a density of 3 × 10^3^ cells/well and cultured overnight at 37 °C in a humidified incubator containing 5% CO_2_ to allow cell attachment. For the cytotoxicity assay, cells were treated with TN-DC at concentrations of 0–200 μg/mL or the individual isoflavones (daidzein, glycitein, and genistein) at 0–50 μM for 48 h. Following treatment, the cells were fixed with 100 μL of 10% (*w/v*) trichloroacetic acid (TCA) at 4 °C for 1 h. The plates were subsequently rinsed gently with deionized water and allowed to air-dry. The fixed cells were stained with 0.054% (*w/v*) sulforhodamine B (SRB) solution for 30 min at room temperature. Excess dye was removed by washing the plates three times with 1% (*v*/*v*) acetic acid, after which the plates were air-dried. The protein-bound SRB dye was solubilized with 100 μL of 10 mM Tris base (pH 10.5), and the absorbance was measured at 540 nm using a microplate reader. Cell viability was expressed as the percentage of the untreated control.

### 4.8. Sample Treatment and UVB-Irradiation

Dermal fibroblast cells were seeded in a 24-well plate at a density of 5 × 10^4^ cells per well and incubated overnight at 37 °C in a humidified atmosphere with 5% CO_2_. The cells were pretreated with TN-DC or the active isoflavones for 6 h prior to UVB irradiation (30 mJ/cm^2^) using a UVB generator (UVP CL-1000 Ultraviolet Crosslinker, Upland, CA, USA) that emits 302 nm UVB radiation. The UV dose was calibrated using a VLX-3W radiometer. After UVB exposure, PBS was removed, and the cells were incubated with 0.5% FBS DMEM containing different concentrations of tested compounds for 48 h. Cell viability was then assessed using the SRB assay.

### 4.9. Apoptosis Evaluation

Apoptosis was assessed using the FITC Annexin V Apoptosis Detection Kit (BioLegend, San Diego, CA, USA; Cat. No. 420201) according to the manufacturer’s instructions. Human dermal fibroblasts were pretreated with TN-DC or the active isoflavones for 6 h prior to UVB irradiation (30 mJ/cm^2^). Following irradiation, the cells were incubated in DMEM containing 0.5% fetal bovine serum (FBS) supplemented with the corresponding treatments for an additional 24 h. The cells were harvested, washed twice with PBS, and resuspended in the supplied binding buffer. The cell suspension was stained with 2.5 μL Annexin V-FITC and 2.5 μL propidium iodide (PI) for 15 min at room temperature in the dark. The stained cells were immediately analyzed using a CytoFLEX flow cytometer (Beckman Coulter, Brea, CA, USA), and the data were processed using CytExpert for DxFLEX version 2.0 software.

### 4.10. Mitochondrial Membrane Potential Quantification

Mitochondrial membrane potential (MMP) was evaluated using the MitoView™ 633 fluorescent probe (Biotium, Fremont, CA, USA) according to the manufacturer’s instructions. Human dermal fibroblasts were pretreated with TN-DC, daidzein, or glycitein for 6 h before UVB irradiation (30 mJ/cm^2^). Following irradiation, the cells were incubated in DMEM containing 0.5% fetal bovine serum (FBS) supplemented with the corresponding treatments for an additional 24 h. After incubation, the cells were harvested and stained with 75 nM MitoView™ 633 prepared in serum-free DMEM for 20 min at 37 °C in a humidified atmosphere containing 5% CO_2_, protected from light. The stained cells were washed once with phosphate-buffered saline (PBS) and immediately analyzed using a DxFLEX flow cytometer (Beckman Coulter, Brea, CA, USA) with 638 nm excitation and 660 nm emission settings. Data were analyzed using CytExpert for DxFLEX Flow Cytometer version 2.5.

### 4.11. Enzyme-Linked Immunosorbent Assay (ELISA)

The secretion of IL-6 and IL-8 was quantified using enzyme-linked immunosorbent assay (ELISA). Human dermal fibroblasts were pretreated with TN-DC, daidzein, or glycitein for 6 h before UVB irradiation (20 mJ/cm^2^) and incubated in DMEM containing 0.5% fetal bovine serum (FBS) supplemented with the corresponding treatments for 18 h. Culture supernatants were then collected, and the concentrations of IL-6 and IL-8 were measured using commercially available ELISA kits (BioLegend, San Diego, CA, USA) according to the manufacturer’s instructions. Absorbance was recorded at 450 nm using a microplate reader, and cytokine concentrations were determined from standard curves generated with the supplied standards.

### 4.12. Collagen Synthesis Assay

Dermal fibroblasts were seeded in 12-well plates for 24 h and incubated at 37 °C in an atmosphere of 5% CO_2_. After incubation, the cells were pre-treated with 0.5% FBS DMEM medium for 24 h. The medium was then removed and pre-treated with various concentrations of TN-DC and isoflavones for 6 h before UVB irradiation (20 mJ/cm^2^). Following irradiation, the cells were incubated in DMEM containing 0.5% fetal bovine serum (FBS) supplemented with the corresponding treatments for an additional 48 h. The culture medium was harvested, and soluble collagen content was quantified using a Sirius Red collagen staining kit (Chondrex Inc., Redmond, WA, USA) according to the manufacturer’s instructions. The resulting collagen pellets were dissolved in extraction buffer, and the absorbance was measured at 540 nm using a microplate reader. Total collagen content was calculated against a standard curve and expressed as a percentage of the untreated control.

### 4.13. Intracellular ROS Production

Intracellular reactive oxygen species (ROS) generation was measured using the 2′,7′-dichlorodihydrofluorescein diacetate (DCF-DA) assay. Human dermal fibroblasts were seeded in 24-well plates at a density of 7.5 × 10^4^ cells/well and incubated overnight to allow cell attachment. The cells were then pretreated with the fractions for 6 h and incubated with DCF-DA for 30 min before being UVB irradiated (at 20 mJ/cm^2^). After incubation for 1 h, fluorescence intensity was measured every 5 min for up to 60 min using a fluorescence microplate reader at an excitation/emission wavelength of 485/525 nm. Intracellular ROS levels were expressed relative to the UVB-irradiated control group.

### 4.14. Western Blot Analysis

Fibroblast cells were seeded at a density of 2 × 10^5^ cells/well in 6-well plates overnight at 37 °C in a humidified atmosphere with 5% CO_2_. Then, the cells were pretreated with different concentrations of TN-DC or standard compounds in serum-free DMEM overnight prior to UVB irradiation. The cells were washed once with 500 µL of PBS, 1000 µL of PBS supplemented with the TN-DC or standard compounds was added, then the cells were irradiated with UVB 30 mJ/cm^2^ using a UVB generator (UVP CL-1000 Ultraviolet Crosslinker, Upland, CA, USA) and further incubated with serum-free DMEM supplemented with TN-DC or standard compounds for 1 h. The cells were harvested by trypsinization and lysed in RIPA buffer. Before loading, total protein quantification was performed using the Bradford assay with Bradford Protein Assay Reagent (Thermo Fisher Scientific, Waltham, MA, USA). An equal amount of protein (approximately 19.5–22.5 µg/lane) was loaded onto an SDS-PAGE gel, and proteins were transferred to a membrane via electroblotting. To verify protein transfer efficiency and assess loading consistency, the membrane was stained post-transfer using Ponceau S. The membrane was then blocked with 2.5% BSA for 1 h before incubation with the primary antibody (diluted in 2.5% BSA) at 4 °C overnight. Following primary antibody incubation, the membrane was washed four times with TBST (5 min per wash) before imaging using the iBright™ FL1500 Imaging System (Thermo Fisher Scientific, Waltham, MA, USA). The band intensity was quantified using ImageJ version 1.54t.

### 4.15. Statistical Analysis

Statistical analyses were performed using GraphPad Prism version 10.2.3 (GraphPad Software, San Diego, CA, USA). Differences among multiple groups were analyzed using one-way analysis of variance (ANOVA) followed by Dunnett’s multiple-comparisons test, with the UVB-treated group serving as the reference control. A *p*-value of <0.05 was considered statistically significant.

## 5. Conclusions

The present study highlights the protective effects of TN-DC and its active isoflavones, daidzein and glycitein, against UVB-induced dermal fibroblast cell apoptosis, inflammation, and collagen depletion. These photoprotective benefits are driven by a robust suppression of intracellular ROS, upregulation of endogenous antioxidant machinery, and the subsequent downregulation of key pro-inflammatory mediators (IL-6, IL-8, COX-2, and iNOS). Mechanistically, the anti-photoaging properties of TN-DC are mediated through the coordinated modulation of the JNK, ERK and Akt signaling cascades. Overall, these findings position this traditional fermented soybean extract as a promising natural candidate for dermatological protection, warranting further in vivo investigations to confirm its therapeutic efficacy.

## Figures and Tables

**Table 1:** Determination of isoflavones in the TN-DC fraction using HPLC. Data are presented as the mean ± SD from three independent experiments (*n* = 3).

Compounds	TN-DC
Daidzin (µg/mg)	ND
Glycitin (µg/mg)	ND
Genistin (µg/mg)	ND
Daidzein (µg/mg)	41.28 ± 5.21
Glycitein (µg/mg)	21.25 ± 9.15
Genistein (µg/mg)	72.57 ± 27.09

ND: Not detected.

**Table 2:** DPPH and ABTS radical scavenging activities of TN-DC and its major isoflavones. Radical scavenging activity is expressed as IC_50_ (mean ± SD), obtained from three independent experiments (*n* = 3).

Compounds	DPPH Radical Scavenging Activity (IC_50_)	ABTS Radical Scavenging Activity (IC_50_)
Vitamin E (mg/mL)	0.003 ± 0.001	ND
Trolox (mg/mL)	ND	0.005 ± 0.00
TN-DC (mg/mL)	5.42 ± 1.5	0.97 ± 0.28
Daidzein (mM)	57.01 ± 41.97	0.21 ± 0.03
Glycitein (mM)	46.24 ± 38.01	0.21 ± 0.04

ND: Not detected.

## Data Availability

Data are contained within the article and Supplementary Material.

## Supplementary Materials

The following supporting information can be downloaded at: https://www.mdpi.com/article/10.3390/ijms27167303/s1.

## References

[1] Lee L.-Y., Liu S.-X. (2022). Pathogenesis of Photoaging in Human Dermal Fibroblasts. Int. J. Dermatol. Venereol..

[2] Cavinato M., Jansen-Dürr P. (2017). Molecular mechanisms of UVB-induced senescence of dermal fibroblasts and its relevance for photoaging of the human skin. Exp. Gerontol..

[3] Wlaschek M., Tantcheva-Poór I., Naderi L., Ma W., Schneider L.A., Razi-Wolf Z., Schüller J., Scharffetter-Kochanek K. (2001). Solar UV irradiation and dermal photoaging. J. Photochem. Photobiol. B.

[4] Britto S.M., Shanthakumari D., Agilan B., Radhiga T., Kanimozhi G., Prasad N.R. (2017). Apigenin prevents ultraviolet-B radiation induced cyclobutane pyrimidine dimers formation in human dermal fibroblasts. Mutat. Res. Genet. Toxicol. Environ. Mutagen..

[5] Katiyar S.K., Matsui M.S., Mukhtar H. (2000). Kinetics of UV Light–induced Cyclobutane Pyrimidine Dimers in Human Skin In Vivo: An Immunohistochemical Analysis of both Epidermis and Dermis. Photochem. Photobiol..

[6] Wondrak G.T., Roberts M.J., Cervantes-Laurean D., Jacobson M.K., Jacobson E.L. (2003). Proteins of the extracellular matrix are sensitizers of photo-oxidative stress in human skin cells. J. Investig. Dermatol..

[7] Tong T., Park J., Moon Y., Kang W., Park T. (2019). α-ionone protects against UVB-induced photoaging in human dermal fibroblasts. Molecules.

[8] Dong K.K., Damaghi N., Picart S.D., Markova N.G., Obayashi K., Okano Y., Masaki H., Grether-Beck S., Krutmann J., Smiles K.A. (2008). UV-induced DNA damage initiates release of MMP-1 in human skin. Exp. Dermatol..

[9] Tanveer M.A., Rashid H., Tasduq S.A. (2023). Molecular basis of skin photoaging and therapeutic interventions by plant-derived natural product ingredients: A comprehensive review. Heliyon.

[10] Yuan X., Li H., Lee J.S., Lee D.H. (2025). Role of Mitochondrial Dysfunction in UV-Induced Photoaging and Skin Cancers. Exp. Dermatol..

[11] Wei M., He X., Liu N., Deng H. (2024). Role of reactive oxygen species in ultraviolet-induced photodamage of the skin. Cell Div..

[12] Wongkarn S., Chewonarin T., Ruangsuriya J., Taya S., Dejkriengkraikul P., Yodkeeree S. (2025). Biochemical Mechanism of Thai Fermented Soybean Extract on UVB-Induced Skin Keratinocyte Damage and Inflammation. Int. J. Mol. Sci..

[13] Fotopoulou A., Angelopoulou M.T., Pratsinis H., Mavrogonatou E., Kletsas D. (2024). A subset of human dermal fibroblasts overexpressing Cockayne syndrome group B protein resist UVB radiation-mediated premature senescence. Aging Cell.

[14] Kim W.O., Kim S.A., Jung Y.A., Suh S.I., Ryoo Y.W. (2020). Ultraviolet b downregulated aquaporin 1 expression via the MEK/ERK pathway in the dermal fibroblasts. Ann. Dermatol..

[15] Plaza M., Pozzo T., Liu J., Gulshan Ara K.Z., Turner C., Nordberg Karlsson E. (2014). Substituent effects on in vitro antioxidizing properties, stability, and solubility in flavonoids. J. Agric. Food Chem..

[16] Tang S., Wang B., Liu X., Xi W., Yue Y., Tan X., Bai J., Huang L. (2025). Structural insights and biological activities of flavonoids: Implications for novel applications. Food Front..

[17] Zielonka J., Gȩbicki J., Grynkiewicz G. (2003). Radical scavenging properties of genistein. Free Radic. Biol. Med..

[18] Bevilacqua M.A., Iovine B., Iannella M.L., Gasparri F., Monfrecola G. (2011). Synergic effect of genistein and daidzein on UVB-induced DNA damage: An effective photoprotective combination. BioMed Res. Int..

[19] Williamson E.M. (2001). Synergy and other interactions in phytomedicines. Phytomedicine.

[20] Caesar L.K., Cech N.B. (2019). Synergy and antagonism in natural product extracts: When 1 + 1 does not equal 2. Nat. Prod. Rep..

[21] Agrawal R., Hu A., Bollag W.B. (2023). The Skin and Inflamm-Aging. Biology.

[22] Hussein R.S., Bin Dayel S., Abahussein O., El-Sherbiny A.A. (2025). Influences on Skin and Intrinsic Aging: Biological, Environmental, and Therapeutic Insights. J. Cosmet. Dermatol..

[23] Liu H., Dong J., Du R., Gao Y., Zhao P. (2024). Collagen study advances for photoaging skin. Photodermatol. Photoimmunol. Photomed..

[24] Briganti S., Picardo M. (2003). Antioxidant activity, lipid peroxidation and skin diseases. What’s new. J. Eur. Acad. Dermatol. Venereol..

[25] Calvo M.J., Navarro C., Durán P., Galan-Freyle N.J., Hernández L.A.P., Pacheco-Londoño L.C., Castelanich D., Bermúdez V., Chacin M. (2024). Antioxidants in Photoaging: From Molecular Insights to Clinical Applications. Int. J. Mol. Sci..

[26] Prior R.L., Wu X., Schaich K. (2005). Standardized methods for the determination of antioxidant capacity and phenolics in foods and dietary supplements. J. Agric. Food Chem..

[27] Forman H.J., Davies K.J.A., Ursini F. (2014). How do nutritional antioxidants really work: Nucleophilic tone and para-hormesis versus free radical scavenging in vivo. Free Radic. Biol. Med..

[28] Zhang M., Hwang E., Lin P., Gao W., Ngo H.T.T., Yi T.H. (2018). *Prunella vulgaris* L. Exerts a Protective Effect Against Extrinsic Aging Through NF-κB, MAPKs, AP-1, and TGF-β/Smad Signaling Pathways in UVB-Aged Normal Human Dermal Fibroblasts. Rejuvenation Res..

[29] Mavrogonatou E., Angelopoulou M., Rizou S.V., Pratsinis H., Gorgoulis V.G., Kletsas D. (2022). Activation of the JNKs/ATM-p53 axis is indispensable for the cytoprotection of dermal fibroblasts exposed to UVB radiation. Cell Death Dis..

[30] Gary A.S., Rochette P.J. (2020). Apoptosis, the only cell death pathway that can be measured in human diploid dermal fibroblasts following lethal UVB irradiation. Sci. Rep..

[31] Kim H., Jang J., Song M.J., Park C.-H., Lee D.H., Lee S.-H., Chung J.H. (2022). Inhibition of matrix metalloproteinase expression by selective clearing of senescent dermal fibroblasts attenuates ultraviolet-induced photoaging. Biomed. Pharmacother..

[32] Liao P.-L., Li C.-H., Chang C.-Y., Lu S.-R., Lin C.-H., Tse L.-S., Cheng Y.-W. (2012). Anti-ageing effects of alpha-naphthoflavone on normal and UVB-irradiated human skin fibroblasts. Exp. Dermatol..

[33] Wisidsri N., Chansriniyom C., Limpanasitikul W. (2025). Protective Effects of Syringaresinol against UVB-Induced Human Dermal Fibroblast Senescence and Apoptosis. Trends Sci..

[34] Ding Y., Jiratchayamaethasakul C., Lee S.H. (2020). Protocatechuic aldehyde attenuates uva-induced photoaging in human dermal fibroblast cells by suppressing mapks/ap-1 and nf-κb signaling pathways. Int. J. Mol. Sci..

[35] Piao M.J., Fernando P.M.D.J., Kang K.A., Fernando P.D.S.M., Herath H.M.U.L., Kim Y.R., Hyun J.W. (2024). Rosmarinic Acid Inhibits Ultraviolet B-Mediated Oxidative Damage via the AKT/ERK-NRF2-GSH Pathway In Vitro and In Vivo. Biomol. Ther..

[36] Re R., Pellegrini N., Proteggente A., Pannala A., Yang M., Rice-Evans C. (1999). Antioxidant activity applying an improved ABTS radical cation decolorization assay. Free Radic. Biol. Med..

[37] Contreras-Guzmán E.S., Strong F.C. (1982). Determination of Tocopherols (Vitamin E) by Reduction of Cupric Ion. J. Assoc. Off. Anal. Chem..

[38] Mapoung S., Umsumarng S., Semmarath W., Arjsri P., Srisawad K., Thippraphan P., Yodkeeree S., Dejkriengkraikul P. (2021). Photoprotective effects of a hyperoside-enriched fraction prepared from houttuynia cordata thunb. On ultraviolet B-induced skin aging in human fibroblasts through the MAPK signaling pathway. Plants.

