# Supplementary material for: Isoflavone-Rich Fraction of Traditional Thai Fermented Soybean (Thua Nao) Protects Dermal Fibroblasts from Photoaging by Modulating MAPK and Akt Signaling Pathways
Source: Int J Mol Sci. 2026 Aug 16;27(16):7303. doi: 10.3390/ijms27167303 (PMC13513677; doi:10.3390/ijms27167303)
Supplement: Supplementary file 1 [file ijms-27-07303-s001.zip › ijms-4462008-supplementary.pdf]

SUPPLEMENTARY DATA

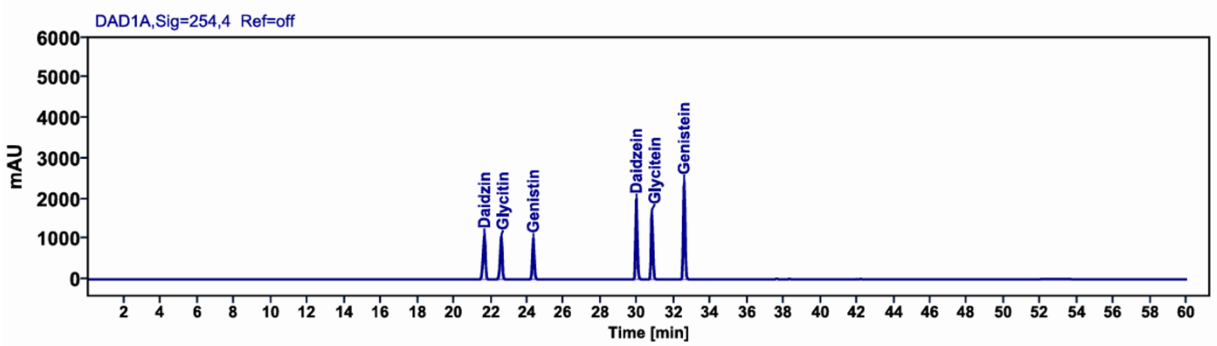

Signal: DAD1A,Sig=254,4 Ref=off

RT [min]	Type	Width [min]	Area	Height	Area%	Compound Concentration	Name
21.650	BB	1.01	9465.24	1129.32	14.17		Daidzin
22.573	BB	1.31	8776.21	1090.76	13.14		Glycitin
24.322	BV	1.34	7454.47	1018.35	11.16		Genistin
29.943	BB	0.87	13166.66	1994.96	19.72		Daidzein
30.791	BV	0.81	11264.17	1732.13	16.87		Glycitein
32.548	BV	2.05	16654.26	2463.34	24.94		Genistein
		**Sum**	**66781.01**				

Supplementary Figure S1 HPLC chromatogram of mixed isoflavone reference standards (250 μg/mL each), including daidzin, glycitin, genistin, daidzein, glycitein, and genistein. Retention times were used for peak identification in TN-DC.

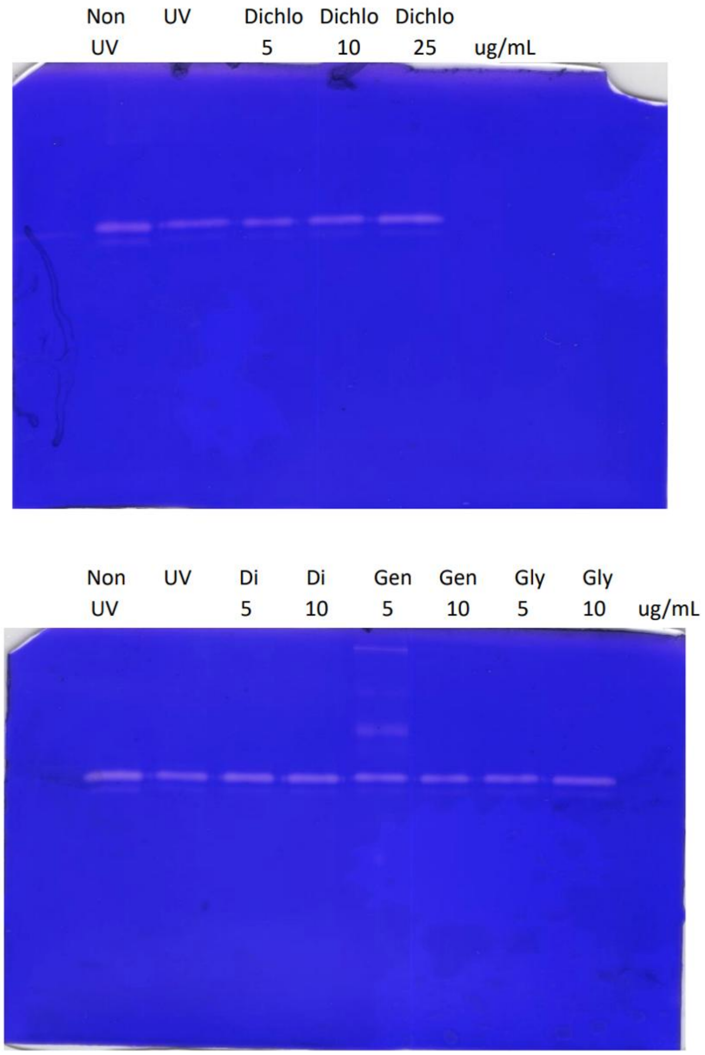

Supplementary Figure S2. Representative gelatin zymograms showing MMP-2 activity in conditioned media collected from UVB-irradiated human dermal fibroblasts treated with TN-DC, daidzein, glycitein and genistein.
